# Editorial: Promoting participation following neurotrauma

**DOI:** 10.3389/fresc.2022.1121684

**Published:** 2023-01-12

**Authors:** Linda Barclay, Jennifer Coker, Feng-Hang Chang

**Affiliations:** ^1^ Department of Occupational Therapy, Monash University, Melbourne, VIC, Australia; ^2^ Research Department, Craig Hospital, Englewood, CO, United States; ^3^ College of Public Health, Taipei Medical University, Taipei, Taiwan

**Keywords:** participation, acquired brain injury (ABI), spinal cord injury (SCI), neurotrauma, social inclusion

**Editorial on the Research Topic** Promoting participation following neurotrauma

Participation in major life areas is one of the main goals of rehabilitation, and a measure of rehabilitation success following neurotrauma such as spinal cord injury (SCI) or acquired brain injury (ABI). Participation includes active engagement at the community level, and allows for social connectedness with other people and communities. The Convention on the Rights of Persons with Disability (1) sets forth the rights of people with disabilities to participate in cultural life, recreation, leisure and sport, have equal access to primary and secondary education, have equal rights to vocational training, adult education, and the right to work and make a living. The importance of participation following an injury or acquired disability is reflected in the participation domains of the International Classification of Functioning Disability and Health (2). These include: Interpersonal Interactions and Relationships (family relationships; intimate relationships; sexual relationships); Major Life Areas (school education; higher education; remunerative employment; economic self-sufficiency); Community, Social and Civic Life (recreation and leisure—physical fitness activities included in this domain; religion and spirituality; human rights). This special topic includes a range of studies that expand understanding of the concept of participation, the influences on it, and interventions aimed at enhancing participation of people in the community following neurotrauma.

In their article, Rajala et al. posit that synthesizing how people with ABI define participation will help identify aspects of participation that is important to them. They conducted a scoping review of qualitative literature to define and characterize participation from the perspective of people with ABI. Six articles were included and synthesized in their review. They then linked the identified themes to the ICF. Their results provide insight into the complexity of the concept participation for people with ABI, providing important information to clinicians and researchers supporting people with ABI.

Two studies investigated factors that influence participation of people following neurotrauma. Chui et al. noted that depression, a common co-morbidity following ABI, impacts rehabilitation outcomes and participation. Their qualitative study found that people with ABI who self-identified as having low mood, valued receiving validation from their healthcare providers and having their concerns seen and believed. The quality of healthcare providers' interactions could have either a positive or negative effect on the patients' recovery expectations. Amsters et al. undertook a literature review and thematic synthesis of qualitative studies relating to, or describing, influences on participation in life after SCI. Their analysis of 24 articles found a range of interconnected elements that influence participation. Among these were the physical and social environment, the physical health and function of the individual with SCI, as well as their psychological state. Most influences were potentially modifiable through relevant rehabilitation approaches.

The resumption of meaningful societal roles is a high priority for people with moderate-severe traumatic brain injury (TBI), but difficult to attain. Understanding changes in social participation of people with TBI over time is needed in order to develop successful interventions. Hart and Rabinowitz examined patterns of change in social participation among people with moderate–severe TBI. For 75% of the participants in their study, social participation did not change between years 1 and 2 post-injury. Changes in Functional Independence Measure (FIM) scores and public vs. private insurance were predictors of change in social participation. A decline in FIM indicated a decline in social participation. The authors recommended that future research should more directly explore the impact of financial factors and other resources on social participation.

Driving is essential for independence, and participation in the community, including for people with ABI (Alhashmi et al.). However, currently there are no guidelines to inform assessment and rehabilitation related to facilitating return to driving following ABI. Alhashmi et al. conducted a systematic review that aimed to understand the assessment methods used internationally to evaluate driving competence among people with ABI. They found four main approaches to driver assessment: neuropsychological test, off-road screening tools, simulator testing and comprehensive driver assessments. However, results revealed a lack of consistency in approaches used.

Interventions for people with ABI were described in three articles in this special topic issue. Walking as a physical activity is recommended as an accessible activity that can improve post-concussion syndrome following ABI. Alarie et al. evaluated an 8 -week walking intervention delivered using telehealth. They found that post-concussion syndrome, anxiety, fatigue and health-related quality of life all improved following completion of the intervention. Quilico et al. describe the participatory and co-design methods used to develop a TBI-Health program aimed at providing an inclusive community-based environment to facilitate exercise and sport participation (Quilico et al.). They found that important physical, psychological, and social benefits were gained by participants in the program. In their article, Coetzer and Ramos describe a neurobehavioral therapy approach to brain injury rehabilitation used in their service, and how it has evolved over time. The effectiveness of this approach was evaluated using retrospective data analysis, which showed improvements across a range of participation areas. Case vignettes were used to illustrate key components of the three clinical stream's (restoration, compensation and scaffolding) approach.

The contributors have explored a range of important issues related to participation in people with disabilities, and the information provided in these articles provide valuable information for clinicians and researchers aiming to support the participation of people who have experienced neurotrauma.

## References

[1] Convention on the Rights of Persons with Disabilities. Available online: https://www.ohchr.org/en/instruments-mechanisms/instruments/convention-rights-persons-disabilities. (Accessed November 21, 2022)

[2] International Classification of Functioning, Disability and Health (ICF). Available from: https://www.who.int/standards/classifications/international-classification-of-functioning-disability-and-health. (Accessed November 21, 2022)

